# Large Daily Stock Variation Is Associated with Cardiovascular Mortality in Two Cities of Guangdong, China

**DOI:** 10.1371/journal.pone.0068417

**Published:** 2013-07-16

**Authors:** Hualiang Lin, Yonghui Zhang, Yanjun Xu, Tao Liu, Jianpeng Xiao, Yuan Luo, Xiaojun Xu, Yanhui He, Wenjun Ma

**Affiliations:** 1 Guangdong Provincial Institute of Public Health, Guangzhou, China; 2 Guangdong Provincial Center for Disease Control and Prevention, Guangzhou, China; Cinvestav-Merida, Mexico

## Abstract

**Objective:**

The current study aimed to examine the effects of daily change of the Shenzhen Stock Exchange Index on cardiovascular mortality in Guangzhou and Taishan, China.

**Methods:**

Daily mortality and stock performance data during 2006–2010 were collected to construct the time series for the two cities. A distributed lag non-linear model was utilized to examine the effect of daily stock index changes on cardiovascular mortality after controlling for potential confounding factors.

**Results:**

We observed a delayed non-linear effect of the stock index change on cardiovascular mortality: both rising and declining of the stock index were associated with increased cardiovascular deaths. In Guangzhou, the 15–25 lag days cumulative relative risk of an 800 index drop was 2.08 (95% CI: 1.38–3.14), and 2.38 (95% CI: 1.31–4.31) for an 800 stock index increase on the cardiovascular mortality, respectively. In Taishan, the cumulative relative risk over 15–25 days lag was 1.65 (95% CI: 1.13–2.42) for an 800 index drop and 2.08 (95% CI: 1.26–3.42) for an 800 index rising, respectively.

**Conclusions:**

Large ups and downs in daily stock index might be important predictor of cardiovascular mortality.

## Introduction

Substantial studies have reported that increased morbidity/mortality of cardiovascular diseases was associated with stressful events, for instance: the US 911 attack [1], earthquakes [2], [3], and large-scale sport events [4], [5]. A large fluctuation in the stock exchange market may also represent important mental and physical stresses that may adversely affect cardiovascular morbidity and mortality, however, to date only a few studies from different areas have investigated the potential impacts of stock market variation on cardiovascular incidence and mortality [6]–[10], with inconsistent findings; furthermore, no multi-city study has been conducted, and the lag pattern of the effect of stock variation on cardiovascular mortality has not been examined yet.

Cardiovascular diseases were one of the leading causes of death in many developed countries, and were listed as the first leading causes of both death and the burden of disease in China in recent years [11]. Cardiovascular diseases accounted for about 32% of total deaths in China in the year of 2005 and an estimated 6.7 million years of productive life among people aged 35–64 years were lost due to cardiovascular diseases in 2000 with a cost of US$30 billion to this country [11], [12].

Unexpected growth and downturn have been witnessed in China’s stock market since 2006 [13], which provided a unique opportunity to explore the impact of large stock variations on human health, particularly cardiovascular mortality. Different from those in developed countries, many of the Chinese stock market investors are new inexperienced individuals with unrealistic expectations about the stock investment, we hypothesize that a large stock market change, especially decrease, has adverse effects on cardiovascular mortality in Chinese population. The current study investigated the association between the Shenzhen stock market variations on the cardiovascular mortality in two cities of Guangdong Province, China.

## Methods

### Settings

Guangzhou, located in southern China, is the capital of Guangdong Province. It has a typical monsoon-influenced climate with wet and hot summers and dry and cool to mild winters. Guangzhou has a population of 11.1 million. The residents of two districts in Guangzhou (Yue Xiu and Li Wan) were selected as the sample for the present study. These districts combined have an area of 92.9 km^2^ and are home to 1.9 million residents [14]. These two districts were chosen for two reasons. Firstly, there were daily air pollution monitoring data available from the two districts, which were collected from three air monitoring stations. Secondly, because most of those living in these two districts were permanent residents; and the mortality data are also of high quality accordingly our previous study [14], [15].

The other city included in this study is Taishan, which is a coastal county-level city, located in the Pearl River Delta, southwest of Jiangmen City (to which it administratively belongs) and 140 kilometers west of Hong Kong. Taishan is a relatively small city with 1 million residents and limited industrial activities, it has a typical monsoon-influenced climate with wet and hot summers and dry and cool to mild winters.

**Figure 1:**
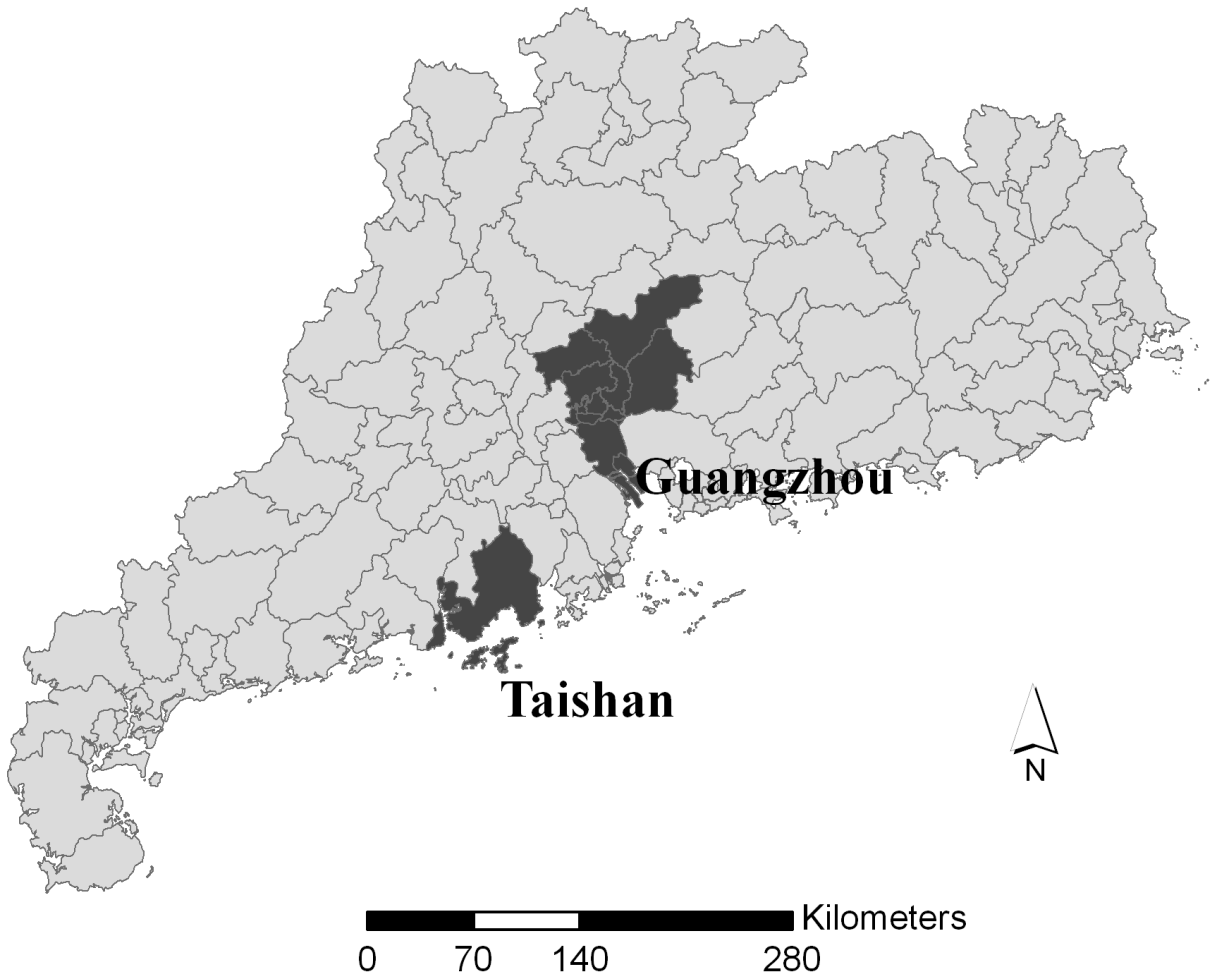
Location of the study area in Guangdong Province, China.

The geographical location of the two cities was illustrated in Figure 1. These two cities were chosen for two reasons. Firstly, Guangzhou was a relatively developed area with many people investing on the stock market, and Taishan was a relatively small and less-developed city with relatively fewer stock investors. Secondly, most of residents in Taishan were Cantonese while there were more migrants in Guangzhou [16]. It was possible that the effects of the stock index change on cardiovascular mortality could be affected by these population characteristics.

### Data Sources

Daily data on counts of deaths from various causes covering the period 2006–2010 were obtained from the Center for Disease Control and Prevention of Guangdong Province. The causes of death were coded according to the 10th revision of the International Classification of Diseases (ICD-10). Cardiovascular disease deaths (ICD-10: I00–I99) were extracted to construct the cardiovascular mortality time series.

Daily stock performance information during the study period, including the opening, closing, change, and percent of change (%) of the Shenzhen stock index was obtained from the Shenzhen Stock Exchange website (http://www.szse.cn). Daily stock index change was defined as the difference between the close index and open index of each day.

Many studies have suggested that ambient air pollution and meteorological factors may affect cardiovascular mortality [17]–[20], so we included these variables as potential confounding factors in the model. Daily ambient air pollution data were obtained from the Environmental Monitoring Center. Air pollution data included particular matter less than or equal to 10 µm in diameter (PM_10_, in ug/m^3^), sulfur dioxide (SO_2_, in ug/m^3^), nitrogen dioxide (NO_2_, in ug/m^3^) and ozone (O_3_, in ug/m^3^). Daily meteorological data for the same period were obtained from Guangdong Meteorological Bureau. The variables included daily mean temperature and relative humidity.

### Statistical Analysis

As count of daily cardiovascular mortality typically followed a Poisson distribution, a time series approach that has been usually used to handle the distributed lag non-linear models was used to examine the effect of stock index change on daily cardiovascular mortality and its associated lag structure [21]. Specifically, we used quasi-likelihood Poisson regression in a generalized linear model to fit the natural logarithm of daily counts of cardiovascular deaths as functions of predictor variables. This model used a “cross-basis” function that evaluated a two-dimensional relationship along the dimensions of stock change and lag days. The method accounted for the over-dispersed Poisson data using the assumption that the total variance was proportional to the total number, with the over-dispersion constant estimated through quasi-likelihood.

We initially constructed a “primary” model and then we did sensitivity analyses to examine the robustness of the effect estimates. The methodology was based on a “cross-basis” function, which allowed the non-linear effect of daily stock index variation at each lag and the nonlinear effects across lag days to be estimated [22]. The “primary” model had a natural cubic spline with 3 df in the lag space and a natural cubic spline with 5 df in the stock index space. We used lags up to 30 days to capture the overall effects according to a previous work [23]. Potential confounders were adjusted for in the model, including an indicator for day of week (DOW), an indicator of public holidays (PH), natural spline for time (6 df/year) in order to control for the seasonal and long-term trend, a smooth function of mean temperature on the current day (Temp_0_, 6 df), a smooth function of the moving average for the previous 3 days’ temperature (Temp_1–3_, 6 df), a smooth function of relative humidity (3 df), and linear function of air pollution (PM_10_, SO_2_, NO_2_ and O_3_). For all of the smooth functions, we used a natural spline basis. The model used for the analysis could be specified:
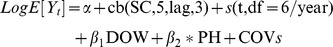
where E(Yt) denotes the expected daily cardiovascular mortality count on day t, cb means the “cross-basis” function, s(·) indicates a smooth function based on natural smooth splines, β is regression coefficient, and COVs are the potential confounding factors.

We reported single day and cumulative effects of extreme stock increase/decrease on cardiovascular mortality along certain lags with 0 change as the reference value.

### Sensitivity Analysis

Because the risk estimates usually vary with the model specifications in time-series analysis [24], [25], we performed additional sensitivity analyses: use of alternative degrees of freedom (4–7 df) for temporal adjustment and changing the degrees of freedom for meteorological variables. We also used the Shanghai Stock Market as the predictor to examine its effect on the cardiovascular mortality.

All statistical analyses were two-sided and values of P<0.05 were considered statistically significant. The dlnm packages [21] in R software Version 2.14.1 (R Development Core Team, 2012) was used to fit all models.

## Results

**Figure 2:**
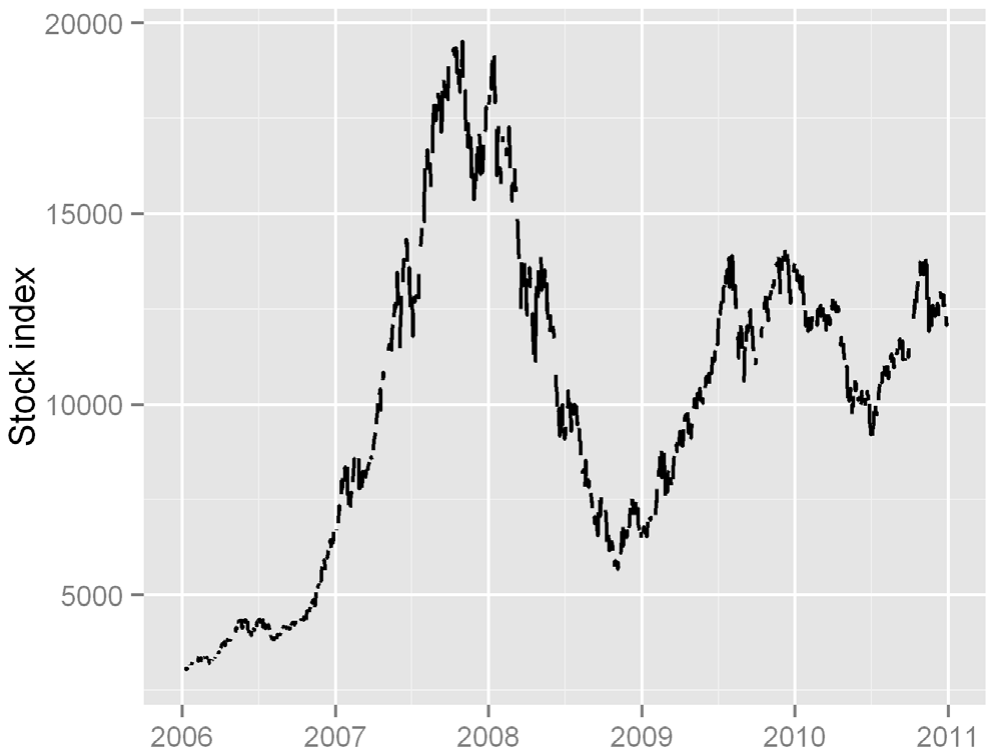
Performance of the Shenzhen Stock Exchange Index, 2006–2010.

Figure 2 illustrated the time-series of daily stock index of Shenzhen Stock Exchange Market, suggesting a drastic fluctuation. During the study period, the stock index surged more than six fold from 2,950 in early 2006 to 19,500 in October 2007, and then declined rapidly to 5,670 in November 2008 before fluctuating around 10,000 in the following time period. Similar temporal fluctuation was also observed in Shanghai Stock Exchange Index.

Table 1 summarized the daily weather conditions, air pollutants, and cardiovascular mortality in the two cities over the study period. The daily stock index variation ranged from −1040.3 to 902.7 with a mean of 11.0 over the study period. There were, on average, 10.9 daily cardiovascular deaths in Guangzhou, and 11.6 in Taishan. The daily mean temperature was similar in the two cities, 22.9°C in Guangzhou and 22.6°C in Taishan. Whereas, higher relative humidity was observed in Taishan (76.6%) than Guangzhou (71.1%). Daily mean concentration of PM_10_, SO_2_, NO_2_ and O_3_ were 72.0, 47.6, 59.3 and 35.0 µg/m^3^ in Guangzhou and 42.8, 27.9, 22.3 and 21.5 µg/m^3^ in Taishan, respectively.

**Table 1:** Summary statistics of daily weather conditions, air pollutants and cardiovascular mortality in Guangzhou and Taishan, China.

	Min	Max	Mean	SD
**Guangzhou**				
Daily stock index change	−1040.3	902.7	11.0	189.9
Cardiovascular mortality	1.0	36.0	10.9	4.2
Mean temperature (°C)	5.4	33.5	22.9	6.2
Relative humidity (%)	25.0	99.0	71.1	13.0
PM_10_ (µg/m^3^)	8.3	268.6	72.0	41.7
SO_2_ (µg/m^3^)	2.4	289.2	47.6	33.3
NO_2_ (µg/m^3^)	13.0	199.4	59.3	32.1
O_3_ (µg/m^3^)	0.1	154.5	35.0	24.7
**Taishan**				
Cardiovascular mortality	1.0	56.0	11.6	4.6
Mean temperature (°C)	5.4	31.6	22.6	5.8
Relative humidity (%)	29.0	99.0	76.6	12.3
PM_10_ (µg/m^3^)	0.0	370	42.8	48.7
SO_2_ (µg/m^3^)	0.0	214.0	27.9	37.5
NO_2_ (µg/m^3^)	0.0	192.0	22.3	27.2
O_3_ (µg/m^3^)	0.0	178.0	21.5	26.8

SD: standard deviation.

**Figure 3:**
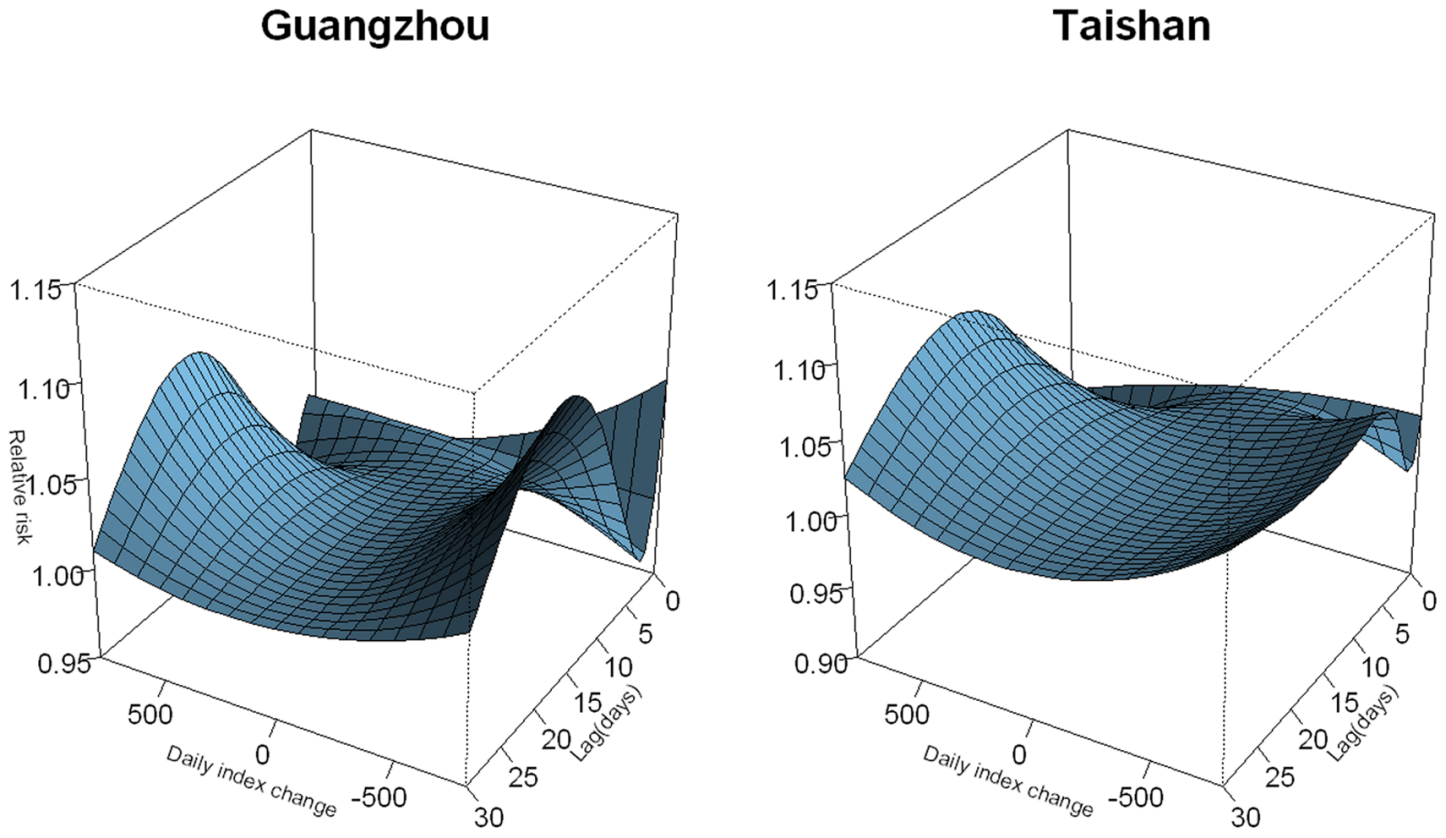
Three-dimension plot of RR along daily index change and lags, with reference at 0 index change.

An overall picture of the effect of daily stock index on cardiovascular mortality in both cities was illustrated in Figure 3, showing a three-dimensional pattern of the relative risk (RR) along daily stock index and lag days. The RR was calculated with none index change as the reference. No significant effects were observed for the first 10 days, afterwards an increased cardiovascular mortality was found for both daily index increase and decrease, and the risk diminished after about 25 days.

**Figure 4:**
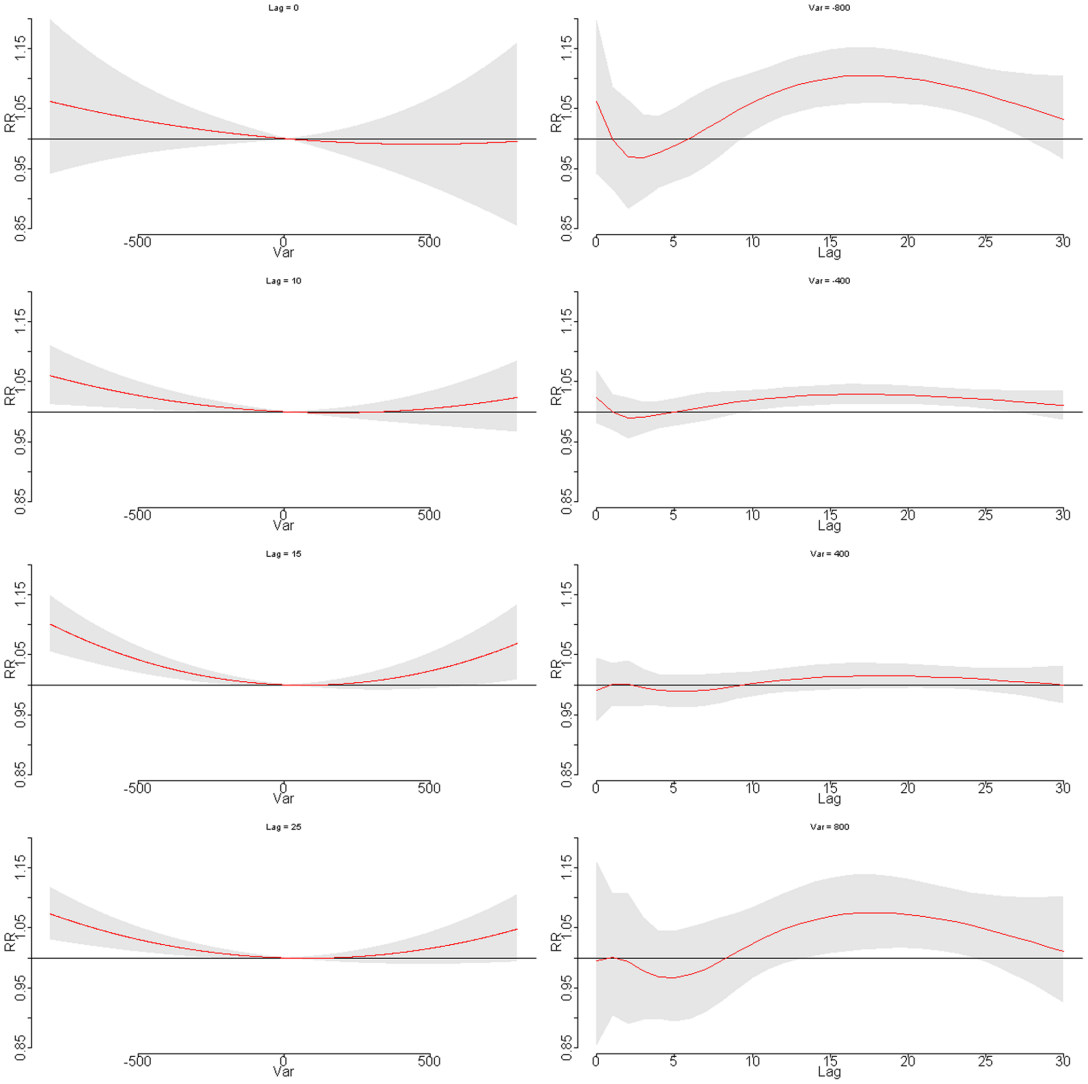
Plot of RR by stock index change at specific lags (left), RR by lag at specific index changes (right) in Guangzhou. Reference at 0 index change.

The relative risk of daily cardiovascular mortality by stock change at specific lag days (0, 10, 15, and 25 days) and by lag at specific index changes (−800, −400, 400, and 800), were plotted for Guangzhou (Figure 4). A U-shape relationship was observed at lag 10–25 days. The index change-mortality relationship appeared to change with delayed days, with non-significant immediate effect in the first 10 days, and it also suggested that extreme index decrease (−800) began to have impact at around 10 days and lasted for about 15 days; on the other hand, a large index rising began to effect at 15th day until 23th day. The result suggested that both stock index rising and declining had delayed adverse impacts on cardiovascular mortality, with index drop having more prominent effect in Guangzhou.

**Figure 5:**
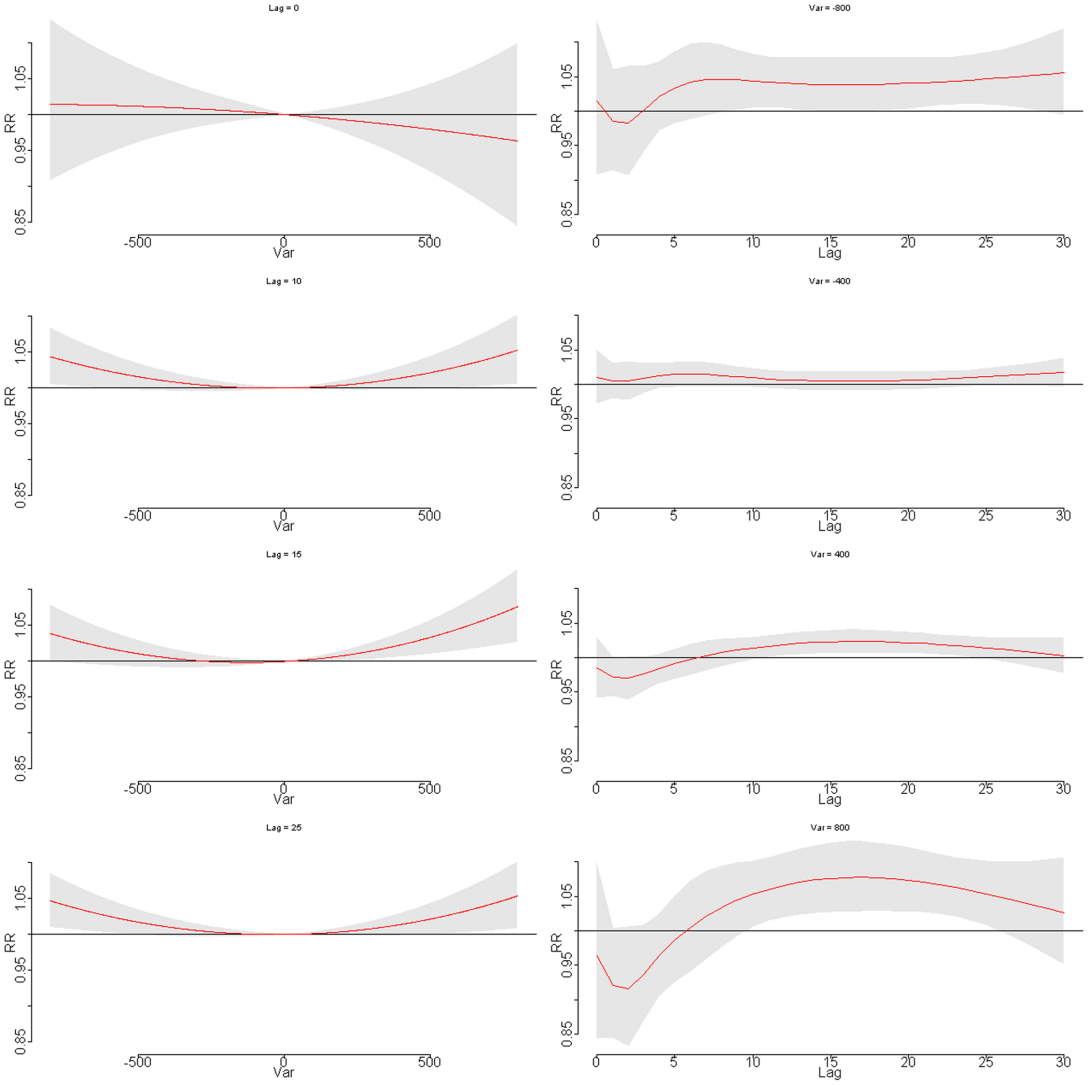
Plot of RR by stock index change at specific lags (left), RR by lag at specific index changes (right) in Taishan. Reference at 0 index change.

The relative risks of daily cardiovascular mortality by daily stock index variation at specific lag days (0, 10, 15, and 25 days) and by lag at specific index changes (−800, −400, 400, and 800) were plotted for Taishan (Figure 5). Similar pattern was observed in Taishan with a U-shape at around 10–25 days. The effects of the stock variation was not statistically significant at the first 10 days, but became harmful in the following lag days, and it appeared that extreme index increase had more serious effect on cardiovascular mortality than the index drop in Taishan.

The single day effect and cumulative effect of the stock index changes on the daily cardiovascular mortality over different lag days were illustrated for Guangzhou in Table 2. The relative risk for an 800 index declining was 1.06 (95% CI: 1.01–1.11) at lag 10 day, 1.10 (95% CI: 1.06–1.15) at lag 15 day, and 1.07 (95% CI: 1.03–1.12) at lag 25 day, respectively; and an 800 index rising was associated with elevated cardiovascular mortality with RR 1.07 (95% CI: 1.01–1.14) at lag 15 day and 1.05 (95% CI: 1.00–1.11) at lag 25 day, respectively. During the first 10 days, no significant effects were detected for both decrease and increase in the stock index; we found a cumulative effect size of 1.77 (95% CI: 1.28–2.44) for lag 10–15 days and 2.08 (95% CI: 1.38–3.14) for lag 15–25 days for an 800 index drop, respectively; and increased risk with a cumulative RR 2.38 (95% CI: 1.31–4.31) for an 800 stock index increase on the cardiovascular mortality was observed over lag 15–25 days.

**Table 2:** Relative risk (RR) and 95% CI in cardiovascular mortality in Guangzhou for specific changes of the Shenzhen Stock Exchange Index at different lag days.

	RR (95% CI)			
	−800	−400	400	800
Lag 0	1.07(0.95–1.20)	1.03(0.98–1.07)	0.99(0.94–1.05)	1.00(0.86–1.17)
Lag 10	1.06(1.01–1.11)	1.02(1.00–1.04)	1.00(0.98–1.02)	1.03(0.97–1.09)
Lag 15	1.10(1.06–1.15)	1.03(1.01–1.04)	1.01(0.99–1.04)	1.07(1.01–1.14)
Lag 25	1.07(1.03–1.12)	1.02(1.01–1.04)	1.01(0.99–1.03)	1.05(1.00–1.11)
Cumlag0–10	1.00(0.99–1.01)	1.00(0.99–1.004)	1.00(0.996–1.002)	1(0.998–1.001)
Cumlag10–15	1.77(1.28–2.44)	1.2(1.07–1.36)	1.02(0.89–1.18)	1.28(0.86–1.90)
Cumlag15–25	2.08(1.38–3.14)	1.18(1.01–1.38)	1.26(1.02–1.56)	2.38(1.31–4.31)

Cum: cumulative.

The single day effect and cumulative effect of the stock index changes on the daily cardiovascular mortality over different lag days were illustrated for Taishan in Table 3. Similar pattern was found in Taishan. Both the index rising and declining were associated with increased cardiovascular mortality at lag 10–25 days. The RR for an 800 index drop was 1.04(95% CI: 1.01–1.08) at lag 10 days, 1.04 (95% CI: 1–1.08) and at lag 15 days and 1.05 (95% CI: 1.01–1.09) at lag 25 days, respectively; while the RR for 800 rising was 1.05 (95% CI: 1.01–1.1) at lag 10 days, 1.08 (95% CI: 1.03–1.13) at lag 15 days, and 1.05 (95% CI: 1.01–1.1) at lag 25 days, respectively. And the cumulative RR over lag 15–25 days was 1.65 (95% CI: 1.13–2.42) for an 800 index declining and 2.08 (95% CI: 1.26–3.42) for an 800 index rising, respectively.

**Table 3:** Relative risk (RR) and 95% CI in cardiovascular mortality in Taishan for specific changes of the Shenzhen Stock Exchange Index at different lag days.

	RR (95% CI)			
	−800	−400	400	800
Lag 0	1.02(0.91–1.13)	1.01(0.97–1.05)	0.99(0.94–1.03)	0.96(0.85–1.10)
Lag 10	1.04(1.01–1.08)	1.01(1.00–1.02)	1.01(1.00–1.03)	1.05(1.01–1.10)
Lag 15	1.04(1.00–1.08)	1.01(0.99–1.02)	1.02(1.01–1.04)	1.08(1.03–1.13)
Lag 25	1.05(1.01–1.09)	1.01(1.00–1.02)	1.01(1.00–1.03)	1.05(1.01–1.10)
Cumlag0–10	1.01(0.68–1.49)	1.04(0.90–1.19)	0.91(0.76–1.08)	0.78(0.47–1.28)
Cumlag10–15	1.06(0.79–1.42)	0.98(0.88–1.08)	1.13(1.00–1.27)	1.40(0.99–1.99)
Cumlag15–25	1.65(1.13–2.42)	1.10(0.96–1.26)	1.23(1.04–1.47)	2.08(1.26–3.42)

Cum: cumulative.

Subgroup analyses were performed to examine which age group had more prominent impact by the stock index variations. For both cities, we found that the observed effects were mainly driven by the older age groups (65 years old and above); it was also found there was relative shorter lay days; the adverse effects in the older age groups began at about 7 lag days (See supplementary Figures S1–S4).

We changed df (4–7) for time to control for seasonality and temporal trend, which gave similar results. We changed df for meteorological factors and various air pollutants, the estimated effects of stock index change were not substantially changed. And similar results were obtained when we used the Shanghai Stock Market Index changes as the exposure variable.

## Discussion

This study showed that large changes in daily stock index were associated with increased cardiovascular mortality in Guangzhou and Taishan, with a delayed effect at about 10 days later; the consistent findings in these two cities suggested that the observed relationship was robust.

Up to date, only four individual studies have examined the effect of stock variations on human health [6]–[9]. In one study, researchers found that increased risk of acute myocardial infarction with cardiac catheterization was linked to increased opening value of NASDAQ Index, using the data from the Duke healthcare system [6]. In Shanghai, investigators [7], [9] found that stock market volatility had an acute impact on coronary heart disease mortality and stroke mortality after controlling for potential confounding factors, and this effect was similar for increase and decrease in the stock market index. And a recent study in Los Angeles did not find any significant effect of the 2008 stock market crash on mortality rate of cardiovascular diseases, after accounting for seasonal variation [8].

Our study found a delayed effect of stock fluctuation on cardiovascular mortality, which was different from the acute effect observed in Shanghai [7]. We believed that the delayed effect cardiovascular was biologically plausible, because the stock market ups and downs could have immediate effect on cardiovascular morbidity (hospitalization or emergency room visits), which might cause daily mortality increase afterwards [26]. However, the hospital admission data were not available for us, which did not allow us to explore it further. On the other hand, the discrepancy between the present study and US might be due to different experiences among different populations. Guangdong Province was among the first area in China to open up to foreign markets, and investment on stock market in Guangdong was several years earlier than other cities in China, and thus stock market investors were relatively experienced than those in other areas and usually had realistic expectations on the financial return. However, they were still not as mature as people in US, where investors usually hired financial advisers to manage their investments and feedback on stock performance was often limited to reading the monthly statement [8].

The mechanism underlying the adverse cardiovascular effect of stock variation remained unclear. However, it has been speculated that emotional and physical stress might have played an important role [7], [27]–[29]. Large changes in the stock markets could be important emotional, psychological, and physical stress that may adversely influence the normal cardiovascular activity [1], [2], [4]. There have been an enormous amount of studies linking psychological stress to cardiovascular health [30]–[35]. For example, after the US September 11 attack, anxiety-related emergency room visits increased dramatically [35], and stress responses to the September 11 attack have been associated with a 53% increased incidence of cardiovascular ailments over the three subsequent years [30]. In another large scale study, stress from work, family and finance was found to be associated with increased risk of acute myocardial infarction [36]. Psychological stress can result in coagulation abnormalities [30], [37] and produce hemoconcentration through stress-induced decreases in plasma volume [38]. Additionally, studies have also suggested that the coronary microcirculation may fail to dilate during acute mental stress [39]. Furthermore, some people with pre-existing cardiovascular illness may exaggerate body responses to the acute stress, including sudden increase of blood pressure, heart rate, and rate of ventricular contraction [40]–[42]. Therefore, it was biologically plausible that psychological stress may increase the risk of cardiovascular mortality, and lead to unexpected deaths among patients with pre-existing cardiovascular conditions.

This study found that the stock index rising had more prominent harmful effects than the index drops in Taishan, and converse results were observed in Guangzhou. The underlying reasons were unclear. Differences in population characteristics among the two cities (e.g. socio-economic status, adaptation to the index change, racial composition) might be possible explanations. The investors in Taishan were generally young and inexperienced, who might have more stressful reaction to the sudden stock rising.

Our study had two major strengths. Firstly, this study investigated the impacts of stock index change on cardiovascular mortality in two cities using an advanced statistical approach (DLNM). The distributed lag non-linear approach can flexibly examine the possible lag and non-linear effects. Although this model was relatively complex and had many parameter specifications, our sensitivity analyses suggested that the results of the study were insensitive to the model specification. Secondly, our study populations were relatively advanced in the stock market experience than others in China, which made our findings a possible mirror of the future picture of population in other Chinese cities.

On the other hand, a few limitations should be considered when interpreting the findings from this study. Firstly, no data were available for the proportion of people investing on the stock market, and its temporal variation in these two cities was not available. Anecdotal evidence suggested that most of the adult residents in the study area had experience of stock investment. Secondly, our analysis was preliminary and exploratory; we could not exclude the possibility that there were unmeasured confounding factors that might be associated with both stock variation and cardiovascular mortality. For example, we did not have the data on PM_2.5_ and carbon monoxide (CO), which have been reported to be related with mortality [43], [44].Thirdly, our study was ecological in nature which did not allow us to explore individual-based association and limited our capacity for causal inference.

In conclusion, our study suggests that large changes in stock index may adversely affect cardiovascular mortality with a 10–25 lag days. Thus, elucidation of the effects of stock variability on the cardiovascular mortality is important for improvement of public health.

## Supporting Information

Figure S1 **Plot of RR by stock index change at specific lags (left), RR by lag at specific index changes (right) in Guangzhou (0–65 years). Reference at 0 index change.**
(TIF)

Figure S2 **Plot of RR by stock index change at specific lags (left), RR by lag at specific index changes (right) in Guangzhou (65 years and older). Reference at 0 index change.**
(TIF)

Figure S3 **Plot of RR by stock index change at specific lags (left), RR by lag at specific index changes (right) in Taishan (0–65 years). Reference at 0 index change.**
(TIF)

Figure S4 **Plot of RR by stock index change at specific lags (left), RR by lag at specific index changes (right) in Taishan (65 years and older). Reference at 0 index change.**
(TIF)
